# Effect of Silane-Treated Pineapple Leaf Fibre and Hemp Fibre on Green Natural Rubber Composites: Interface and Mechanics

**DOI:** 10.3390/polym18010047

**Published:** 2025-12-24

**Authors:** Siriwan Jansinak, Kwanchai Buaksuntear, Arnaud Spangenberg, Antoine Le Duigou, Darshil U. Shah, Karine Mougin, Wirasak Smitthipong

**Affiliations:** 1 Specialized Center of Rubber and Polymer Materials in Agriculture and Industry (RPM), Department of Materials Science, Faculty of Science, Kasetsart University, Bangkok 10900, Thailand; siriwan.jan@ku.th (S.J.); kwanchai.bu@ku.th (K.B.); 2 Institute of Materials Science of Mulhouse, French National Centre for Scientific Research (CNRS)—University Mixed Research Unit (UMR 7361), University of Haute-Alsace, F-68057 Mulhouse, France; arnaud.spangenberg@uha.fr; 3 Dupuy de Lôme Research Institute (IRDL), French National Centre for Scientific Research (CNRS)—University Mixed Research Unit (UMR 6027), Bionics Group, University of South Brittany, F-56100 Lorient, France; antoine.le-duigou@univ-ubs.fr; 4 Centre for Natural Material Innovation, Faculty of Architecture, University of Cambridge, Cambridge CB2 1PX, UK; dus20@cam.ac.uk; 5 Hub of Talents in Natural Rubber, National Research Council of Thailand (NRCT), Bangkok 10900, Thailand

**Keywords:** sandwich natural rubber composite, pineapple leaf fibre, hemp fibre, Silane69

## Abstract

This study developed a natural rubber (NR) composite reinforced with surface-modified pineapple leaf fibres (PALFs) and hemp fibres (HFs) using a layer-by-layer (sandwich-like) fabrication method. The objectives were to increase the utilisation of the natural fibres as reinforcing agents and to investigate the impact of silane fibre surface modification on the properties of the sandwich composites. Fibre surface characterisation was performed using Fourier transform infrared spectroscopy (FTIR), X-ray photoelectron spectroscopy (XPS), and X-ray diffraction (XRD) to confirm the presence of functional groups from silane and cellulose. The wettability and adhesion properties of the modified fibres were also evaluated. The mechanical properties were investigated via single-fibre tensile tests. Composites with 50 phr silane-treated PALF showed the best compromise in terms of interface adhesion (48.3 mJ/m^2^) and tensile strength (6 MPa). This result was also supported by scanning electron microscopy (SEM), which revealed the absence of voids between the fibres and the NR matrix. Furthermore, dynamic mechanical analysis showed that the PALF composite treated with silane at 50 phr exhibited the best viscoelastic behaviour. NR composites with 50 phr silane-treated PALF have mechanical properties suitable for potential applications in engineering products.

## 1. Introduction

Natural rubber (NR) comprises the majority of the cis-1,4-polyisoprene polymer [1,2]. NR is a highly useful elastomer due to its significant tensile and tear strength, high elongation, and good processability [3,4,5,6]. Additionally, it can be processed into widely used products, such as tyres, inner tubes, and car parts, as well as rubber gloves, belts, bands, and sheets, among other applications [7,8]. However, these products often require the modification of NR with chemicals and reinforcing fillers, such as silica, carbon black, and calcium carbonate, to improve material properties for the intended product use [9]. Apart from synthetic fillers and reinforcements, natural materials can also serve as effective reinforcing agents to produce fully green natural fibre/natural rubber composites.

In recent years, there has been an increasing focus on natural fibre composites because of their environmentally friendly characteristics [10,11]. In addition, their impressive stiffness-to-weight and strength-to-weight ratios, lower cost, and lower carbon emissions have propelled their use in diverse applications, including the automotive and construction sectors [12,13]. Natural fibres can be derived from minerals, animals, and plants [7,14], with plant fibres being most abundant and (therefore) commonly used for natural fibre composites. Amongst plant fibres, bast fibres like flax and hemp are popular due to their excellent mechanical properties [15,16], but secondary fibres (by-products of a utilisation) such as coconut coir fibres, date palm leaf fibres, and pineapple leaf fibres can also be used as low-cost, locally sourced fillers in natural fibre composites. However, due to the generally polar surfaces of plant fibres, they do not adhere well to NR and polyolefinic matrices, and their surface may need to be modified to improve adhesion and, therefore, stress transfer between the matrix and the fibre in the composite. This can be achieved through physical surface modification or chemical surface modification [17,18].

In the literature reviews, the results of PALF surface pretreatment using sodium hydroxide (NaOH) and NaOH/silane revealed that the surface morphology of both untreated and NaOH-treated PALFs exhibited increased roughness due to the partial elimination of hemicellulose, lignin, and other soluble components. In contrast, the surface of silane-treated fibres appeared smoother and more uniform than that of the untreated and NaOH-treated fibres, owing to the modification of surface constituents following alkali and silane treatments. This result is consistent with the study of Valášek et al., who reported that untreated coir and abaca fibres exhibited surface irregularities, while alkali treatment effectively removed surface residues and impurities [19]. Furthermore, silane-treated and alkali-treated fibre-reinforced composites showed better mechanical qualities than fibre-reinforced composites that had not been treated [14]. The effects of three different surface treatments (NaOH, triethoxy(ethyl)silane, and NaOH-silane) of kenaf fibre and PALF on the mechanical properties and strength of their rubber matrix were assessed [20]. The silane treatment of kenaf fibre and PALF produced composites with the highest tensile strength. This occurred as a result of a strong interfacial link between the fibres and matrix. Assessment of the impact of diverse surface treatments regarding the performance of jute fibre-reinforced NR composites revealed the establishment of a silicon–oxygen crosslinking bond in the alkali/silane-treated jute fibres [21]. The torque difference was greater for NR composites filled with alkali/silane-treated jute fibres. A combined alkali/silane treatment resulted in a more intact NR matrix with better jute fibre interaction. In earlier research on the reinforcement effects of PALF and aramid fibre in NR, the PALF was surface-modified using Silane69 before being compounded with NR to produce composites. At low temperatures, the NR composites reinforced with PALF and aramid fibre exhibited significantly higher storage moduli, indicating increased material stiffness. As the temperature was increased, the moduli remained constant until about −50 °C. At higher temperatures, it decreased. This behaviour reflects the reinforcing effect of the fibres, particularly at higher fibre contents, resulting in greater moduli than for unreinforced rubber. Additionally, the tan δ values were consistent with the glass transition temperature (Tg) of NR, and the tan δ peak decreased at greater fibre contents. These findings suggest that both PALF and aramid fibres contribute to improved mechanical strength, especially in the temperature range where the molecular chains of rubber begin to mobilise [22].

Previous research suggests that the production of NR/natural fibre composites requires mixing. However, it is difficult to produce high-fibre-content composites with such an approach. Therefore, we designed a process for preparing high natural-fibre-content NR composites using a fibre layer and an NR layer. This effectively improved the green composite’s mechanical properties. In the current study, an investigation was conducted on the filling of NR with optimal proportions of HF and PALF with and without fibre surface modification. Additionally, we designed a forming technique to produce green composites with a sandwich-like structure, with a layer of natural fibre between two layers of crosslinked NR. This research is applicable to the construction engineering sector and the construction industry.

## 2. Materials and Methods

### 2.1. Materials

Natural rubber (NR), grade STR 20, was supplied by the Rubber Authority of Thailand (Bangkok, Thailand). The chemicals used to produce the NR composites included zinc oxide, stearic acid, carbon black (N330), N-cyclohexyl-2-benzothiazole sulphenamide (CBS), and sulphur, supplied by Siam United Rubber Company Limited (Nakhon Pathom, Thailand). Bis[3-(triethoxysilyl)propyl]tetrasulfide (Silane69) was supplied by Bangkok Metropolis Motor Company Limited (Samut Sakhorn, Thailand). Sodium hydroxide was purchased from Sigma-Aldrich (St. Louis, MO, USA), and ethanol was obtained from RCI Labscan (Bangkok, Thailand).

Pineapple leaf fibre (PALF) was supplied by Rak Banrou Songkhla Limited Partnership (Songkhla, Thailand). Hemp fibre (HF) was obtained from DD Nature Craft Company Limited (Bangkok, Thailand). The characteristics related to the physical and mechanical aspects of both fibres are presented in Table 1. PALF and HF properties differ significantly, especially in terms of density and mechanical properties. PALF is denser than HF, which influences the fibre preparation process for NR composites. PALF tends to exhibit higher tensile strength and modulus than HF. This can be ascribed to the elevated cellulose content in PALF, which is consistent with previous work [23].

### 2.2. Preparation of NR Compounds (Without Natural Fibre)

An internal mixer (MX300, CHAREON TUT Company, Samut Prakan, Thailand) was used to prepare the rubber compounds at rotor speeds between 50 and 60 rpm. Subsequently, we incorporated rubber, the activator group, and carbon black N330 and then blended the mixture for about 10 min (Table 2). Subsequently, we transferred the rubber compound to a two-roll mill (ML-D6L12, Chareon Tut Co., Ltd., Samut Prakan, Thailand) and then incorporated an accelerator and sulphur into the compound, with mixing for 9 min at room temperature.

### 2.3. Natural Fibre Surface Modification

#### 2.3.1. Alkali Treatment

**Figure 1:**
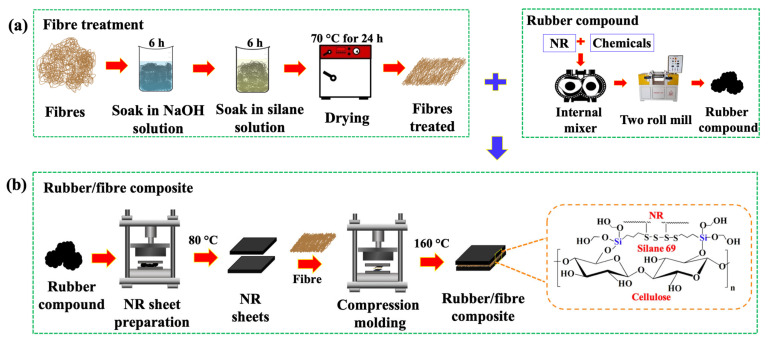
The fabrication of natural rubber/natural fibre sandwich composite samples: (**a**) the fibre treatment process for PALF or HF and the processing of the rubber compound, and (**b**) the preparation of rubber/fibre composite via compression moulding process for the Silane69 cellulosic fibre-treated NR composites.

To modify the fibre surfaces, a solution was prepared by dissolving 5 g of NaOH in 500 mL of deionised water. PALF or HF was submerged in an NaOH solution at room temperature for 6 h. After treatment, the fibres were thoroughly washed with deionised water until all residual NaOH was removed. This was confirmed when the pH of the rinse water returned to neutral, indicating the absence of alkalinity. Subsequently, the fibres were oven-dried at 70 °C for 24 h to eliminate residual moisture and impurities, as illustrated in Figure 1a. Since NaOH is a strong base, it can break chemical bonds in the hemicellulose structure, such as ester and glycosidic bonds, leading to dissolution. The hydroxide ions (OH^−^) cleave these bonds and separate the polysaccharide chains of hemicellulose from the main cellulose fibres, resulting in the breakdown and dissolution of hemicellulose in water [24,25].

#### 2.3.2. Silane Treatment

Firstly, a 10% solution of Silane69 was prepared in 500 mL of ethanol [26]. The natural fibres were then immersed in this solution at room temperature for 6 h. Following this treatment, the fibres were washed many times with ethanol to eliminate the remaining unreacted silane coupling agent. They were subsequently dehydrated in an oven at 40 °C for 6 h, as depicted in Figure 1a. Fibre surface modification can enhance its adhesion capability to the NR. Silane treatment employs a silane coupling agent as an intermediary to establish a chemical linkage between the inorganic fibre surface and the organic polymer matrix. The primary objective is to enhance the interfacial adhesion (interfacial shear strength) between the fibre and the matrix [27,28]. Finally, both treated and untreated fibres, as well as the rubber composites, must be stored in a desiccator to prevent contact with moisture in the air.

### 2.4. Preparation of NR/Natural Fibre Composites

A 1 mm sheet of compound rubber was produced in a compression moulding machine (PR1D-W300L350-PM-HMI, Chareon Tut Co., Ltd., Samut Prakan, Thailand) operated at 80 °C. The NR sandwich composite was moulded using natural fibre, either PALF or HF, in various ratios (0, 25, 50, and 75 phr at equal volume ratios for each fibre). It was placed between two NR sheets as shown in Figure 1b and Table S1. Each specimen was then processed in a compression moulding machine at 160 °C and 1100 psi for vulcanisation. The final composite thickness was fixed at ca. 1 mm. The Silane69 chemically links to the double bonds of the NR compound via sulphur atoms on the Silane69 structure at 160 °C.

### 2.5. Sample Characterisation

#### 2.5.1. Characterisations of PALF and HF

A microscope (Model DSX500, Olympus Corporation, Tokyo, Japan) was used to measure the diameter and length of PALFs and HFs. The surface characteristics of 3–10 specimens of PALF and HF samples were analysed using Fourier transform infrared (FTIR) spectroscopy (Vertex 70, Bruker, Billerica, MA, USA) utilising the attenuated total reflection (ATR) mode, with a germanium crystal as the supporting material. FTIR spectra were recorded over the range of 4000–400 cm^−1^. Moreover, the examination of the surface morphology of the fibres was conducted using scanning electron microscopy (SEM, FEI, Quanta 450 FEI, Eindhoven, the Netherlands). Additionally, surface chemical characterisation of natural fibres was performed using X-ray photoelectron spectroscopy (XPS, SES-2002, VG SCIENTA, Scienta Omicron, Taunusstein, Germany). X-ray diffraction (XRD, STADI-P model, STOE & Cie GmbH, Darmstadt, Germany) was employed to determine the crystalline structure of both treated and untreated PALF and HF. The wettability of NR and fibres was investigated with contact angle measurements utilising a Processor Tensiometer K12 (Krüss, Hamburg, Germany). This measures the static contact angles between a liquid and a solid having a known wetted area and calculates the surface energy of fibres from the Owens, Wendt, Rabel, and Kaelble (OWRK) method using Equation (1) [29]. By substituting this expression into Young’s equation (Equation (2)) [30], the polar and dispersive components of the solid’s surface energy can be extracted from a regression line of the appropriate plot.

(1) $$\gamma_{L}\;*\;\left(1+cos\;\theta \right)=2\sqrt{\gamma_{L}^{d}\;*\gamma_{S}^{d}}+2\sqrt{\gamma_{L}^{p}*\gamma_{S}^{p}}$$

where $\gamma_{L}$ is the surface tension (liquid), θ is the contact angle, $\gamma_{L}^{d}$ is the dispersive component of the surface tension (liquid), $\gamma_{L}^{p}$ is the polar component of the surface tension (liquid), $\gamma_{S}^{d}$ is the dispersive component of the surface energy (solid), and $\gamma_{S}^{p}$ is the polar component of the surface energy (solid) [31].

(2) $${\;\gamma }_{S}=\gamma_{L}\;cos\;\theta +\gamma_{SL}$$

where $\gamma_{S}$ is the surface free energy (solid), and $\gamma_{SL}$ is the interfacial tension between the solid and liquid.

#### 2.5.2. Characterisation of Rubber Compound

The crosslinked rubber compound was characterised at 160 °C according to ISO 6502:2025 [32]. A moving die rheometer (MDR, CGM Technology, Pathum Thani, Thailand) was employed to measure the curing time of the NR compound.

#### 2.5.3. Characterisations of NR/Natural Fibre Composites

Adhesion between two substances results from interatomic and intermolecular forces that are manifest at the contact interface, contingent upon the closeness of the material surfaces [33]. The work of adhesion (W) of the composite was presented in an earlier work [7].

The samples were tested using a universal testing machine (UTM, Shimadzu, AGS-X, Kyoto, Japan) for the assessment of mechanical properties of rubber composites, following the ISO 37:2024 Type 2 standard [34] and mechanical property testing; 3–5 specimens/sample were used [35]. A single-fibre pullout test was applied to characterise the rubber/fibre interfacial interaction, following the methodology outlined in [36]. Briefly, an individual fibre longer than 40 mm was utilised in a test between two rubber sheets measuring 20 mm × 20 mm × 1 mm and subjected to hot pressing at 160 °C. The fibre was maintained in a straight position and threaded through a specially designed frame measuring 20 mm × 20 mm × 1 mm, which included a 1 mm diameter aperture, with a fibre protrusion length of 10–15 mm. The analysis of single-fibre samples inside rubber sheets was conducted in a pullout test utilising this universal testing machine. The specimen under examination exhibited a break at the fibre/composite border that enabled the determination of rubber/fibre interactions.

The dynamic properties of the rubber composite were investigated using a dynamic mechanical analyser (DMA1, Mettler Toledo, Columbus, OH, USA), with specimens prepared at dimensions of 1 mm × 20 mm (width × length). The storage modulus, loss modulus, and loss factor (tan δ) of rubber composite samples were analysed. Tests in single cantilever mode were performed in a temperature scanning mode ranging from −80 °C to 80 °C at 10 °C min^−1^. Further measurements were performed in a shear sandwich mode with a 0 to 25% strain sweep at 25 °C.

A thermogravimetric analyser (TGA, Mettler-Toledo, TGA/DSC 3+, Greifensee, Switzerland) was used in combination with a gas-phase mid-infrared FTIR spectrometer (Bruker, INVENIO^®^ S, Ettlingen, Germany). Samples with dimensions of 2 mm × 2 mm × 1 mm were prepared for the analysis. We scanned rubber composites from 30 to 700 °C at 10 °C·min^−1^ under a nitrogen atmosphere to analyse weight loss and confirm the silane peak.

A correlation between stress (σ) and tensile extension limit (λ) from the tensile test for each rubber composite sample was developed using the Mooney–Rivlin Equation (Equation (3)), where $C_{1}$ and $C_{2}$ represented constant values [37].

(3) $$\frac{\sigma }{\lambda -\frac{1}{\lambda^{2}}}={2C}_{1}+2C_{2}\frac{1}{\lambda }$$

## 3. Results and Discussion

### 3.1. Surface Characteristics of PALF and HF

The diameters of PALFs and HFs with and without Silane69 coating are shown in Figure S1. The uncoated fibres were observed to have larger diameters than the treated fibres. After the Silane69 coating, a decrease in fibre diameter was observed at all measurement points. This suggests that some unwanted surface materials were removed during the surface treatment process. Consequently, the surface texture of the fibres is expected to have a significant influence on rubber compound formation [38,39].

**Figure 2:**
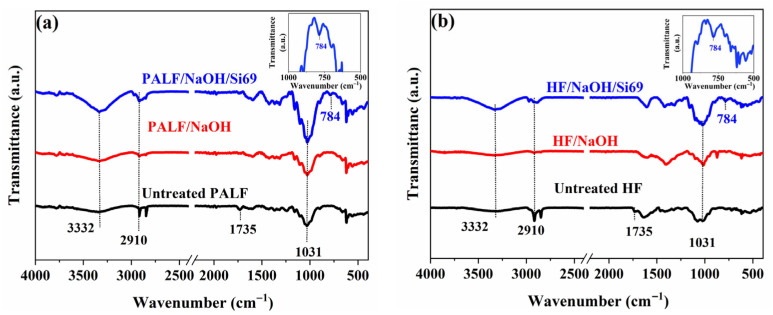
FTIR spectra of natural fibres: (**a**) PALF and (**b**) HF with/without Silane69 treatment.

FTIR analysis with and without silane treatment revealed the functional groups or chemical components of PALF and HF. These results are shown in Figure 2. The untreated PALF and HF (Figure 2a,b, black line) exhibited hemicellulose and cellulose at 2910 cm^−1^ (representing C–H stretching of hemicellulose), 1735 cm^−1^ (representing C=O stretching of hemicellulose), and 3332 cm^−1^ (representing O–H stretching) [40]. The peak characteristics at wavenumbers 2910 cm^−1^ and 1735 cm^−1^ were reduced after alkali treatment (Figure 2a,b, shown as red lines) due to the removal of NaOH impurities [40,41,42]. This agrees with the results of Moonart et al. After cleaning the HF surfaces, HF treated with NaOH could effectively remove surface contaminants compared to untreated hemp fibres [40]. Masud et al. chemically studied the surface modification of PALF using NaOH. It was found to effectively eliminate surface impurities while also enhancing the interfacial adhesion properties of the fibre surfaces [14]. After Silane69 treatment of the PALF and HF surfaces, the silane characteristic appeared at 784 cm^−1^ (shown in Figure 2a,b as blue lines), indicating the bending vibration of siloxane groups (Si–C) on fibre surfaces [43]. These results align with the work of Masud et al., who investigated the surface modification of PALF using Silane69. Their study observed characteristic absorption bands at 700 and 765 cm^−1^, which were attributed to Si–O–Si and Si–C bonding, respectively [14].

**Figure 3:**
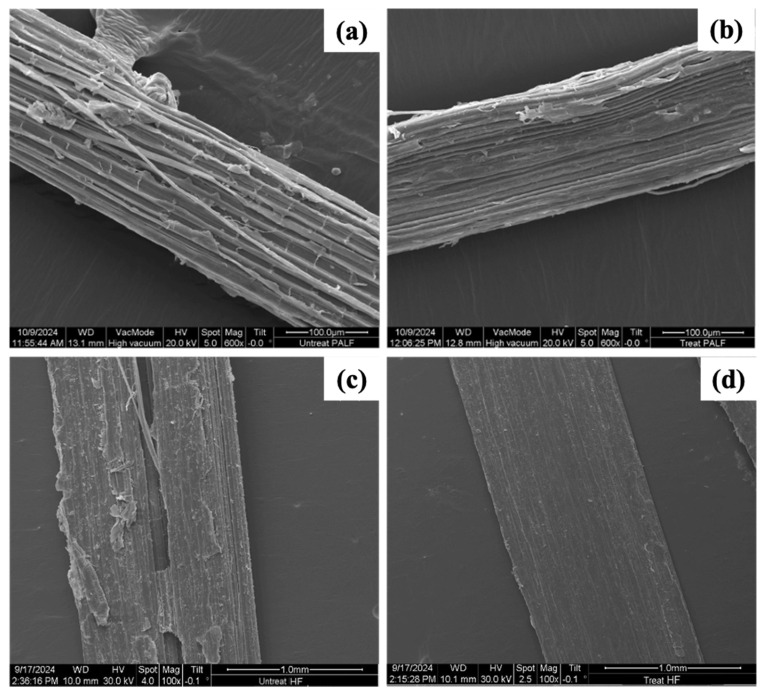
SEM images of a bundle of natural fibres: (**a**) untreated PALF, (**b**) NaOH- and Silane69-treated PALF, (**c**) untreated HF, and (**d**) NaOH- and Silane69-treated HF.

The morphology of both PALF and HF with and without Silane69 is shown in Figure 3 and Figure S4. The untreated PALF and HF (Figure 3a,c). The width of a single PALF and HF was approximately 180 and 550 microns, respectively. The length of a single PALF and HF was approximately 10 and 30 cm, respectively. Additionally, they exhibited a rough surface texture due to the presence of hemicellulose or impurities on the fibre surfaces. After the NaOH and Silane69 treatment (Figure 3b,d), the fibre surfaces exhibited significantly greater smoothness. NaOH successfully removed impurities from the fibre surfaces [14], confirmed by the FTIR results discussed earlier. A treated fibre surface provides for strong adhesion to the matrix [40]. Najeeb et al. revealed that silane surface treatment of PALF led to a smoother surface morphology compared to untreated fibres [44]. Alao et al. studied surface modification of hemp fibres using alkali and silane [45]. They observed that fibres treated with alkali alone, as well as those treated with a combination of alkali and silane, exhibited cleaner and more purified surfaces than untreated fibres. Surface contaminants, including wax and lignin, were eliminated. Moreover, the surface becomes free from impurities (e.g., wax) and chemically more amenable for adhesion. To ensure the effective surface modification of natural fibres, silane treatment was performed using various concentrations. Initially, we applied 2, 5, and 7% silane solutions to the fibres. However, tests using FTIR and XPS did not show any silanol functional groups on the fibre surfaces after these treatments. This absence indicated that the silane concentration was insufficient to form detectable silanol bonds on the surface. Then, the silane concentration was increased to 10%. At this level, we could clearly see the specific signals of silanol groups, which showed that the fibre surfaces were successfully treated. Therefore, a 10% silane solution was chosen to treat the PALF and HF surfaces, which were then used to strengthen natural rubber composites, as this concentration enhanced the attachment strength between the fibres and the NR matrix.

**Figure 4:**
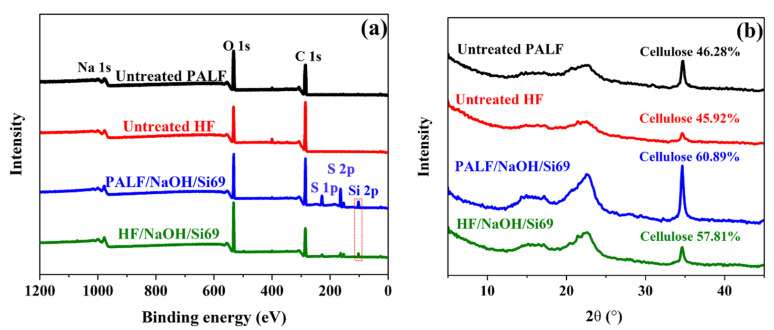
(**a**) Surface analysis of PALF and HF based on the XPS result in which the dotted box presents Si 2p, and (**b**) crystalline structure study of PALF and HF with and without NaOH and Silane69 treatment based on the XRD result.

X-ray photoelectron spectroscopy (XPS) was used to analyse the components and chemical composition of material surfaces, including the elemental composition and their quantities, chemical structures, types of chemical bonds, and oxidation states of atoms. For this work, we compared surface-modified and unmodified PALF and HF, as shown in Figure 4a. The spectra indicated that all samples exhibited peaks at binding energies around 285 and 532 eV, consisting of the primary components, C 1s and O 1s [46]. However, a binding energy peak at 102 eV was detected in the S 1p, S 2p, and Si 2p regions of both surface-modified PALF and HF. This is a distinct feature that can be used to differentiate between surface-modified and unmodified fibres. This peak is believed to have been formed by the silane treatment during the surface modification process, as the molecular structure of Silane69 contains both sulphur and silicon atoms. It was therefore confirmed that sulphur-containing silane was successfully bonded to the surface of the natural fibres.

The XRD patterns of PALFs and HFs with and without Silane69 treatment are shown in Figure 4b. All samples exhibited prominent diffraction peaks at 2θ values of 15.7°, 23.3°, and 34.6°, which correspond to the ($1\bar{1}0$), (200), and (004) crystallographic planes of cellulose, respectively. These results agree with the findings of Lorwanishpaisarn et al., who found that surface-modified cellulose nanocrystals (CNCs) treated with silane exhibited major peaks at 2θ values of 15.5°, 16.3°, 22.2°, and 34.6°, corresponding to the ($1\bar{1}0$), (110), (200), and (004) planes, respectively [47]. These diffraction peaks confirm the presence of crystalline cellulose in both untreated and treated samples.

Understanding the significant adhesion mechanisms is essential for the effective adhesion of multi-component materials [7]. Thus, the wettability properties of PALF and HF, both with and without Silane69 treatment, were analysed and are presented in Table 3. The use of both polar (water) and non-polar (1,2,3-trichloropropane, TCP) liquids was employed to evaluate the wettability of green compounds and natural fibres. Contact angle measurements of at least 90 degrees were observed in water for PALF and HF treated with silane (Table 3). This indicated that a hydrophobic surface character was successfully achieved. NR, a non-polar hydrocarbon compound, is inherently hydrophobic. In the case of silane-treated fibres, the silane structure contains amino groups that form a hydrophobic layer on the surface, thereby preventing water penetration. In terms of surface free energy, PALF and HF treated with silane exhibited slightly higher surface free energies compared to their untreated counterparts. An increase in surface free energy, especially in the dispersive part, enhances interfacial adhesion and compatibility with the rubber matrix [48].

### 3.2. Curing Time of NR Compounds

The curing characteristics of NR compounds at 160 °C were evaluated. The scorch time, defined as the time at which vulcanisation begins, was 1.54 min, while the optimum curing time (t_c90_) was 3.24 min.

### 3.3. Thermal and Interfacial Properties of NR/Natural Fibre Composites

**Figure 5:**
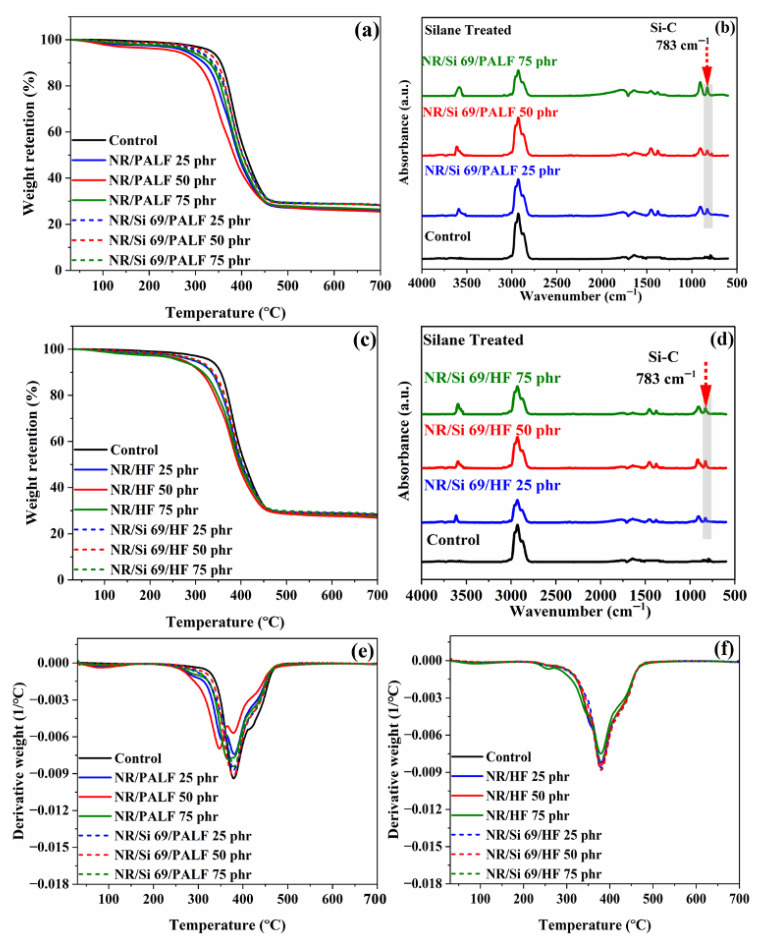
TGA-FTIR curves of rubber composites: (**a**) weight retention of rubber composites with PALF, (**b**) FTIR spectra in the gas phase of rubber composites with treated PALF, (**c**) weight retention of rubber composites with HF, (**d**) FTIR spectra in the gas phase of rubber composites with treated HF, (**e**) derivative weight of rubber composites with PALF, (**f**) derivative weight of rubber composites with HF.

Thermal characteristics of the NR composites with and without silane treatment were investigated using thermogravimetric analysis (TGA). These results are shown in Figure 5. The derivative weights of NR composites with PALF and HF are presented in Figure 5e,f, respectively. The first decomposition occurs close to 86 °C, attributed to water loss and the low thermal stability of volatile materials in the NR composites with PALF and HF (Figure 5a,e). Decomposition of cellulose is responsible for the second event, which happens near 260 °C. The last event occurs at 318 °C and 347 °C, attributed to lignin degradation for samples with untreated natural fibre [49]. For all NR composite samples and unfilled compounded rubber, NR decomposition occurs at 390–400 °C [50]. An approximate 30% residue remained in every sample, as shown in Figure 5a,e.

In the TGA-FTIR analysis, evolved gases were collected during the TGA process and subsequently transferred to a mid-FTIR spectrometer for functional group identification from their spectra. As shown in Figure 5b,d, the FTIR spectra of the NR composite reinforced with PALFs and HFs revealed an Si–C absorption peak at 783 cm^−1^ in the gas phase [51]. This peak was observed in the composites whose fibre surfaces had been modified with silane due to the presence of silicon and carbon atoms in the silane structure. The gas-phase FTIR spectrum was consistent with the solid-phase spectra presented in Figure 2a,b, where a similar Si–C peak appeared at 784 cm^−1^.

The DSC thermograms of the rubber composites reinforced with PALF and HF are shown in Figure S2a,b, respectively. As can be observed, the glass transition temperatures (Tg) of all samples were similar, within the approximate −63 to −60 °C range, dominated by NR. This consistency in Tg values can only be ascribed to the mobility of the rubber molecular chains.

**Figure 6:**
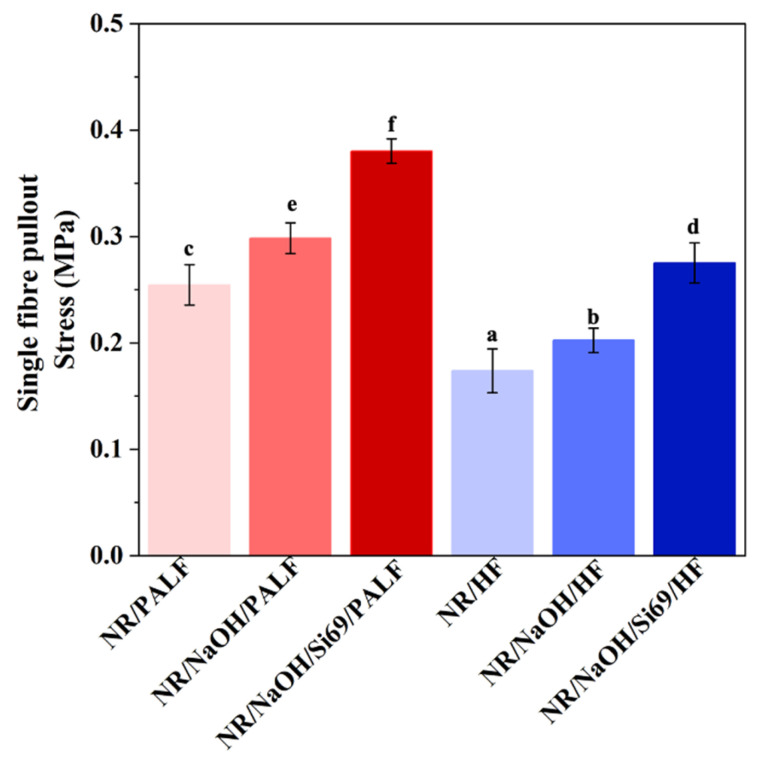
Single-fibre pullout stress of PALF and HF in the rubber matrix. ^a–f^: The one-way analysis indicates statistically significant differences in single-fibre pullout stress among groups with different letters at a 95% significance level (*p* < 0.05).

The fibre–matrix adhesion properties of NR composites were evaluated through single-fibre pullout tests using PALFs and HFs, both with and without silane surface treatment. These results are presented in Figure 6. Incorporation of silane-treated PALF (0.38 MPa) and HF (0.27 MPa) and fibres treated with NaOH-PALF (0.29 MPa) and HF (0.20 MPa) resulted in higher interfacial stress values compared to their untreated counterparts (0.25 MPa for untreated PALF and 0.17 MPa for untreated HF). Considering the fibre breakage characteristics, the protruding fibres broke off near the rubber–fibre interface, while no fibre detachment from the rubber matrix was observed. Therefore, this enhancement is attributed to improved fibre–matrix adhesion facilitated by silane modification. In contrast, the untreated fibres were found to retain surface impurities such as hemicellulose, lignin, and wax, which reduced surface energy and consequently weakened the interfacial adhesion between the fibres and rubber matrix. These results align with those of Asim et al., who reported that PALF possesses a waxy surface layer that lowers surface tension and adversely affects adhesion to the polymer matrix [52]. Moreover, the silane treatment functioned as a coupling agent, improving the adhesion at the interface between the rubber matrix and fibres. PALF-reinforced composites had superior interfacial stress values than those reinforced with HF, signifying a more efficient stress transfer at the fibre/matrix interface. This behaviour was clearly evident in the silane-modified fibre-filled rubber composites, indicating strong rubber–fibre interfacial interaction.

**Figure 7:**
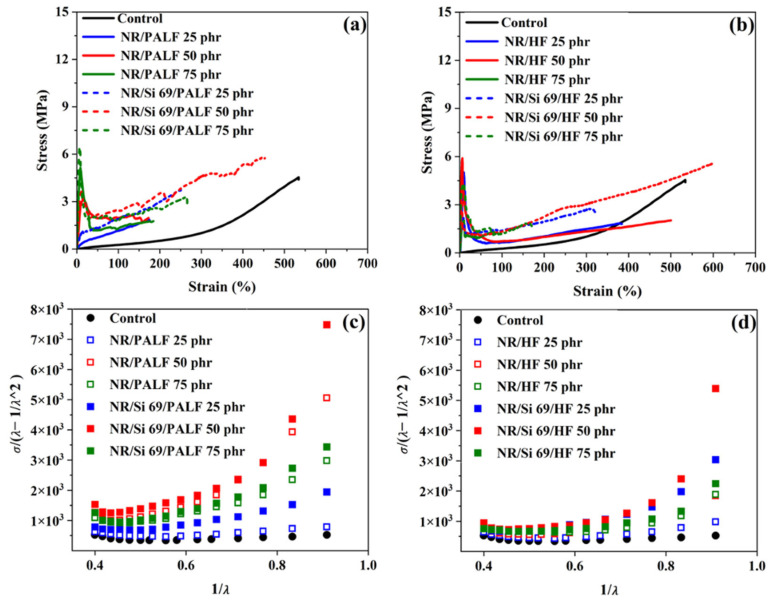
The mechanical properties of NR composites with PALF and HF: (**a**) stress–strain of rubber composites with PALF, (**b**) stress–strain of rubber composites with HF, (**c**) Mooney–Rivlin of rubber composites with PALF, and (**d**) Mooney–Rivlin of rubber composites with HF.

The stress–strain curves of NR composites with PALF and HF are shown in Figure 7a,b, respectively. During the first phase, NR composites exhibited low strain but significantly higher stress or slope values compared to the control formulation due to reinforcement with natural fibres, which improved their stiffness [53,54], as shown in Figure S3a–d and Table S3. The stress in NR composites decreased at a certain stage due to fibre debonding. Approximately 80% of strain-induced stress is due to strain-induced crystallisation of natural rubber [22,53]. Additionally, NR composites (>3.00 MPa) had higher Young’s moduli as their fibre content increased compared to the control NR compound (0.35 MPa). NR composites filled with surface-modified fibres had higher stress–strain values than those filled with unmodified fibres. This is consistent with the Young’s modulus values shown in Figure S3e. Sulphur atoms in Silane69 groups bonded to the horsetail, which aided in NR molecule crosslinking. Natural fibres modified with silane surfaces can promote their adhesion to NR [55]. However, when considering the amount of surface-modified PALF and HF added, it was found that with 50 phr, the strain was comparable to the control formulation; moreover, it had the highest stress value, which might have been because it is a most suitable level for these NR composites.

**Figure 8:**
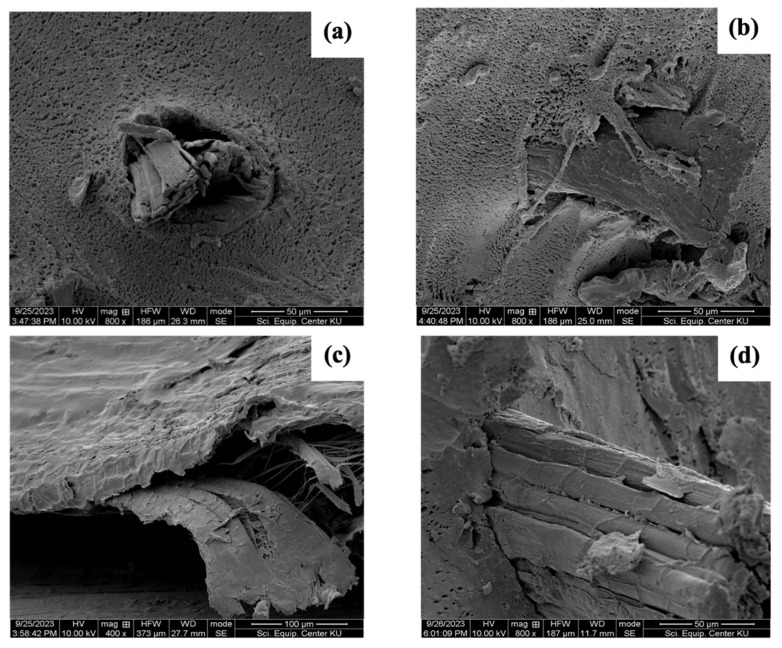
Fractured surface of rubber composites with PALF and HF at 50 phr after tensile strength testing: (**a**) untreated PALF, (**b**) PALF treated with NaOH and Silane69, (**c**) untreated HF, and (**d**) HF treated with NaOH and Silane69.

In SEM imagery, the fracture surface of the NR composites is shown with 50 phr of PALF and HF after tensile strength testing (Figure 8 and Figure S5). PALF and HF pullouts from the NR matrix created many voids [56,57], as shown in Figure 8a,c, respectively. However, silane-treated PALF and HF at 50 phr, shown in Figure 8b,d, respectively, presented no voids, indicating good adhesion between the fibres and NR [40]. This supports observations that samples with the highest mechanical property values yielded the best results. The Mooney–Rivlin curves of rubber composites reinforced with PALF and HF were plotted and are shown in Figure 7c,d, respectively. The C_1_ values tended to increase with greater fibre contents for both PALF and HF (Table S2). When fibre loading was considered, the highest C_1_ values were found at 50 phr, which may be attributed to this being the optimal content for reinforcement. Since 2C_1_ is related to the shear modulus (G′), the increased G can be attributed to the formation of a crosslinked network between the rubber molecular chains and the silane-treated fibre surfaces. This network formation led to an enhanced shear modulus. Additionally, C_2_ was correlated with the entanglement density of the samples, which increased with the development of the network structure depicted in Table S2 [58].

The work of adhesion to rubber composites reinforced with PALF and HF is presented in Table 4. Surface-modified fibres exhibited higher work of adhesion than those with no surface treatment. This effect was particularly evident for PALF-reinforced rubber composites, which showed greater adhesion values than those reinforced with HF. The improvement is attributed to the surface modification process, which increased the dispersive energy of the natural fibres, thereby enhancing their adhesion to the rubber matrix [44]. Furthermore, it was observed that the surface-treated PALF-reinforced rubber composite seemed to have the highest work of adhesion with an uncertainty of 5%. A higher work of adhesion is associated with stronger interfacial bonding [59]. These findings are consistent with the mechanical, thermal, dynamic, and morphological properties of rubber composites reinforced with surface-treated PALF.

**Figure 9:**
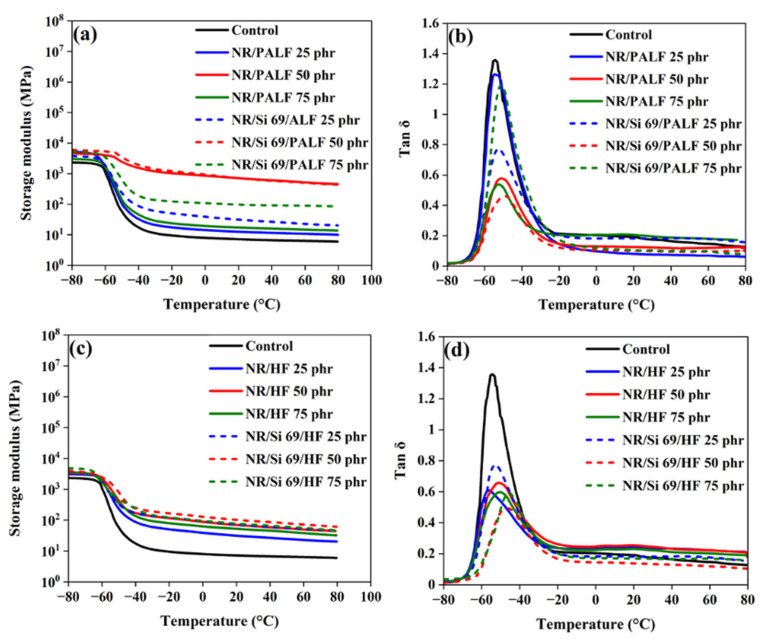
DMA (single cantilever mode) of rubber composites: (**a**) storage moduli of rubber composites with PALF, (**b**) tan δ of rubber composites with PALF, (**c**) storage modulus of rubber composites with HF, and (**d**) tan δ of rubber composites with HF.

The dynamic mechanical characteristics of NR composites in the single cantilever mode with and without silane treatment are shown in Figure 9. They were measured over a −80 °C to 80 °C temperature range. The storage moduli of NR composites with PALF and HF are shown in Figure 9a,c, respectively. In the glassy state, all the composites exhibited similar and high storage moduli [7,60]. As in the stress–strain curves, the Young’s modulus of the NR composites was higher than that of the control formulation. Silane-modified PALF and HF had higher storage moduli than their unmodified versions over a wide temperature range [61]. Additionally, in the rubbery state, the highest storage moduli were obtained when surface-modified PALFs and HFs at 50 phr were used as reinforcements. This could be attributed to the excellent interaction that was established between the surfaces of the natural fibres and the rubber matrix, as well as to the efficient stress transfer that was achieved from the matrix to the fibrous filler [62].

**Figure 10:**
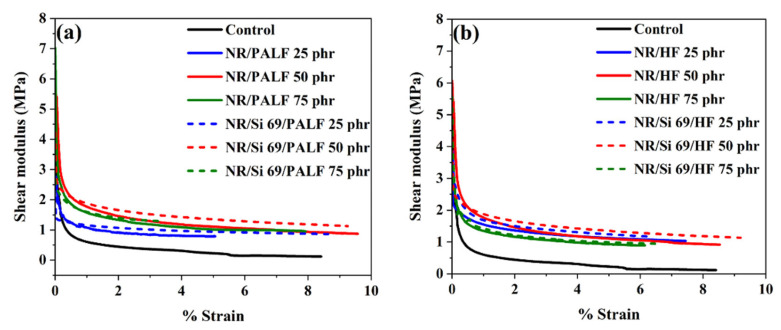
DMA (shear mode) in rubber composite sandwich: (**a**) NR/PALF composites and (**b**) NR/HF composites.

The properties of the rubber composite sandwich structure under shear mode are shown in Figure 10. The obtained results are consistent with those observed in the single cantilever mode. NR composites reinforced with silane-treated PALF and HF exhibited higher shear moduli than those reinforced with untreated fibres. Additionally, the fibre loading at 50 phr was optimal for enhancing the shear modulus of NR composites. These findings suggest that natural fibres can be effectively used as reinforcing materials, and further improvements in the composite properties can be achieved through fibre surface modification [63,64].

## 4. Conclusions

In this study, pineapple leaf fibre (PALF) and hemp fibre (HF) were surface-modified by silane coupling agents to improve interfacial adhesion with the rubber matrix in sandwich-structured composites. The treatment altered the fibres’ wettability, imparting hydrophobicity due to amino-functional silane layers, and increased surface free energy, indicating better matrix bonding. Mechanical properties were significantly enhanced by the treatment. Single-fibre pullout tests showed higher interfacial stress in silane-treated composites, and stress–strain curves exhibited increased stress at low strains, consistent with higher stiffness and an increased Young’s modulus at greater fibre loading. Dynamic mechanical analysis revealed higher storage moduli in all composites, with the 50 phr silane-treated PALF system achieving the best performance, including the highest stress values, improved Mooney–Rivlin parameters, and the strongest interfacial bonding. Overall, the 50 phr silane-treated PALF composite was identified as the optimal formulation for developing sustainable, high-performance bio-based composites.

## Figures and Tables

**Table 1:** Physical and mechanical characteristics of PALF and HF in the form of bundles of fibres.

Properties	PALF	HF
Density (g/cm^3^)	1.07	0.86
Diameter (µm)	200–250	500–600
Length (mm)	10–40	10–60
Tensile strength (MPa)	280–320	190–290
Young’s modulus (GPa)	20–35	14–23
Elongation at break (%)	1–2.5	1–1.5

**Table 2:** Formulation of a rubber compound with no natural fibres.

Ingredient	Content (phr ^1^)
NR STR20	100
Steric acid	1
Zinc oxide	3
Carbon Black N330	40
CBS	1
Sulphur	2

^1^ phr is parts per hundred of rubber.

**Table 3:** Contact angle values and surface energy values of PALF and HF with and without Silane69 treatment.

Sample	Green Compound	Untreated PALF	Treated PALF	Untreated HF	Treated HF
Contact angle with water (°)	96 ± 1 ^b^	87 ± 1 ^a^	101 ± 1 ^d^	87 ± 1 ^a^	108 ± 1 ^c^
Contact angle with TCP (°)	47 ± 1 ^a^	69 ± 1 ^d^	68 ± 1 ^e^	51 ± 1 ^b^	61 ± 1 ^c^
*γ^d^* (mJ/m^2^)	27.65 ^e^	16.44 ^b^	18.63 ^d^	15.52 ^a^	17.34 ^c^
*γ^p^* (mJ/m^2^)	1.42 ^a^	4.84 ^c^	5.01 ^b^	4.75 ^b^	4.89 ^d^
*γ* (mJ/m^2^)	29.08 ^e^	21.28 ^b^	23.64 ^d^	20.28 ^a^	22.23 ^c^

^a–e^: The one-way analysis indicates statistically significant differences in contact angle values and surface energy among groups with different letters at a 95% significance level (*p* < 0.05).

**Table 4:** Work of adhesion of NR composites reinforced with PALF and HF at 50 phr with and without silane treatment.

Work of Adhesion	Untreated PALF	Treated PALF	Untreated HF	Treated HF
*W* (mJ/m^2^) ± 5%	49.7	51.5	47.7	50.0

## Data Availability

The original contributions presented in this study are included in the article/Supplementary Materials. Further inquiries can be directed to the corresponding authors.

## Supplementary Materials

The following supporting information can be downloaded at https://www.mdpi.com/article/10.3390/polym18010047/s1, Figure S1: Natural fibre diameter under optical microscope: (a) untreated PALF, (b) treated PALF, (c) untreated HF, and (d) treated HF; Figure S2: DSC thermograms of rubber composites: (a) with PALF and (b) with HF; Figure S3: The mechanical properties of rubber composites with PALF and HF: (a) stress–strain of rubber composites with untreated PALF, (b) stress–strain of rubber composites with treated PALF, (c) stress–strain of rubber composites with untreated HF, (d) stress–strain of rubber composites with treated HF, and (e) Young’s modulus of rubber composites; Figure S4: SEM images of bundle of natural fibres: (a) untreated PALF, (b) NaOH- and Silane69-treated PALF, (c) untreated HF, and (d) NaOH- and Silane69-treated HF; Figure S5. Fractured surface of rubber composites with PALF and HF at 50 phr after tensile strength testing: (a) untreated PALF, (b) PALF treated with NaOH and Silane69 (c) untreated HF, and (d) HF treated with NaOH and Silane69; Table S1: Formulation of rubber compound with and without natural fibres; Table S2: The Mooney–Rivlin constants (C1 and C2) of the sample; Table S3: The tensile properties of the sample.
