# Supplementary material for: Effect of Silane-Treated Pineapple Leaf Fibre and Hemp Fibre on Green Natural Rubber Composites: Interface and Mechanics
Source: Polymers (Basel). 2025 Dec 24;18(1):47. doi: 10.3390/polym18010047 (PMC12787481; doi:10.3390/polym18010047)
Supplement: Supplementary file 1 [file polymers-18-00047-s001.zip › polymers-4028905-supplementary.pdf]

Supplementary Materials

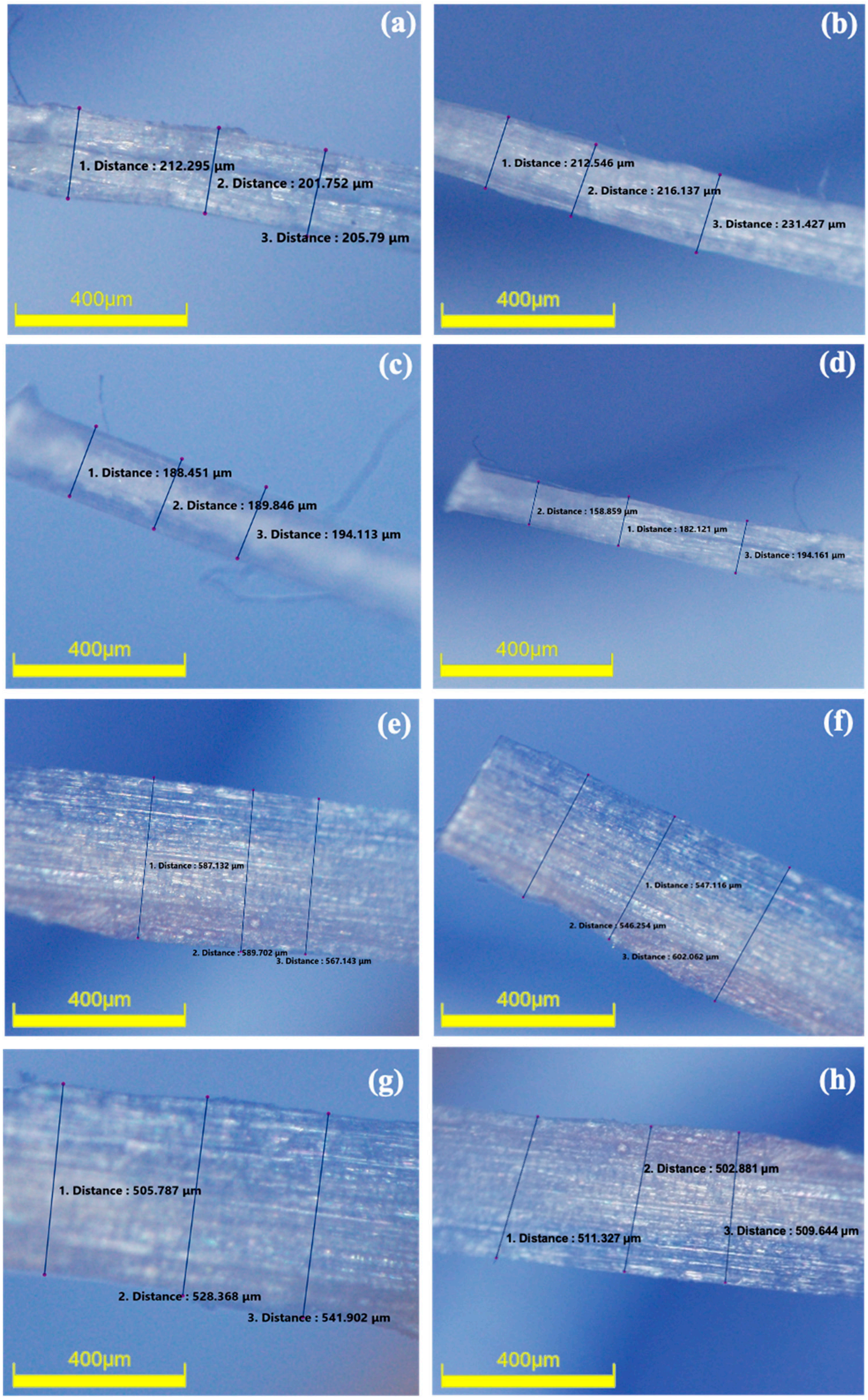

Figure S1. Natural fibres diameter under optical microscope: (**a-b**) untreated PALF, (**c-d**) treated PALF, and (**e-f**) untreated HF and (**g-h**) treated HF.

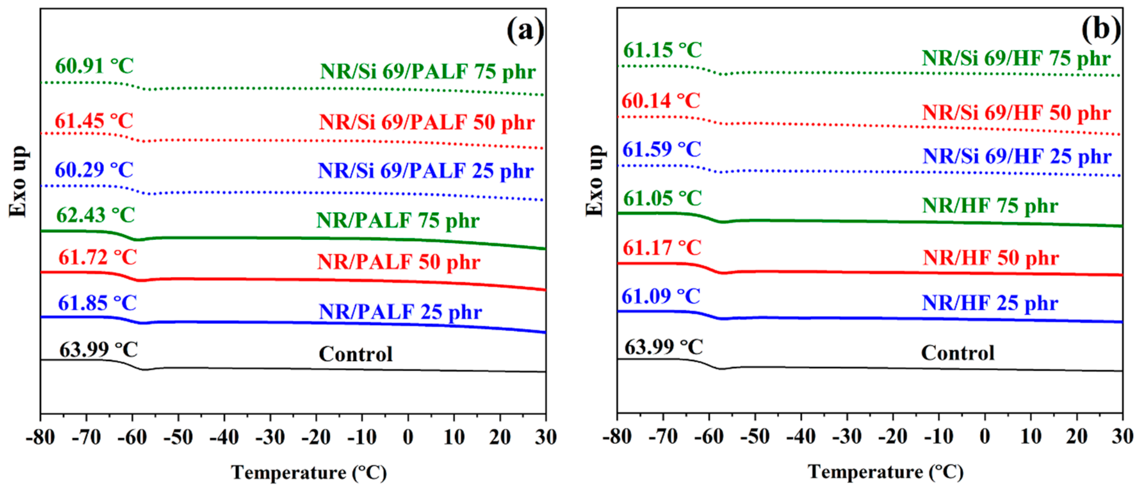

Figure S2. DSC thermograms of rubber composites: (**a**) with PALF and (**b**) with HF.

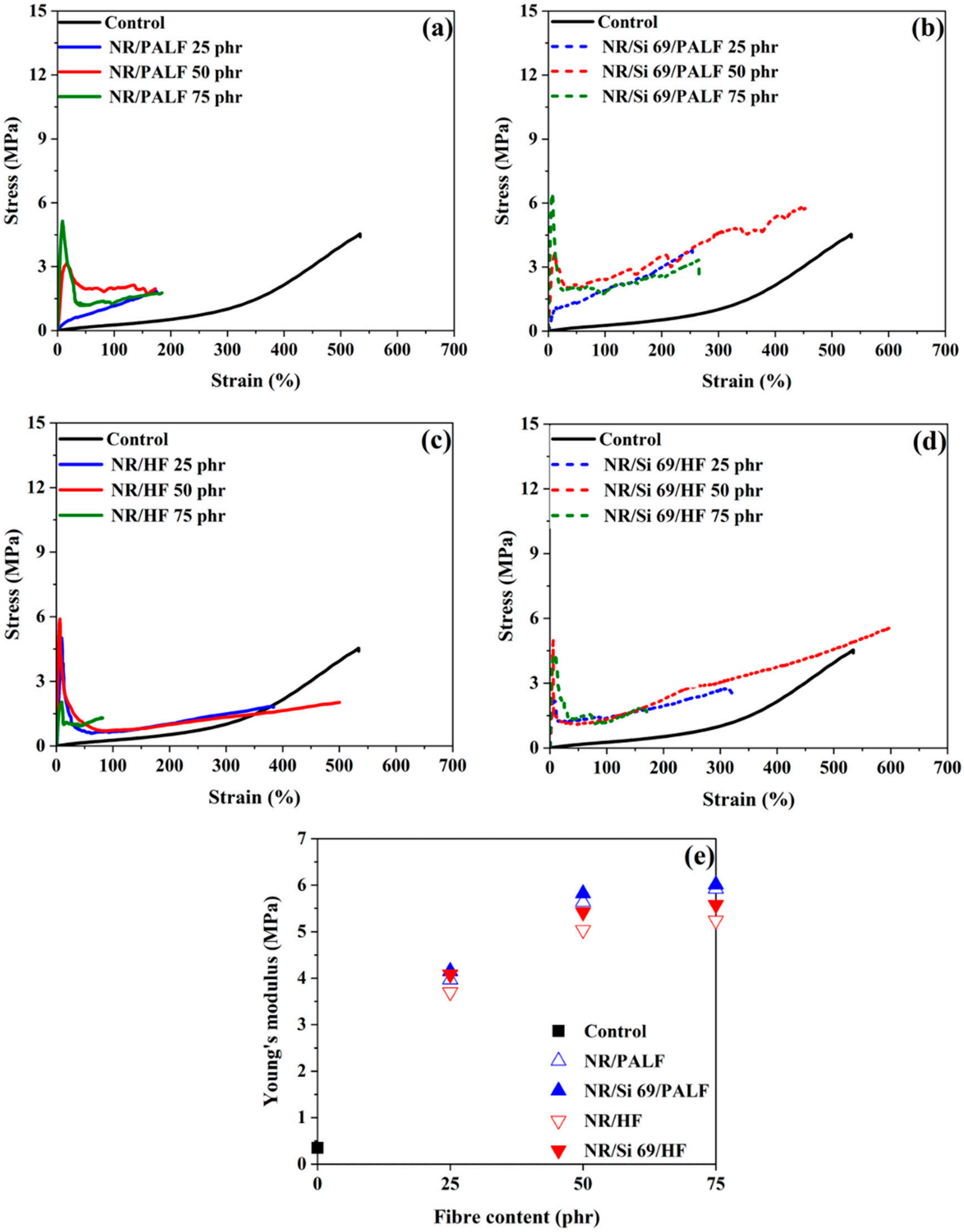

Figure S3. The mechanical properties of rubber composites with PALF and HF: (**a**) stress-strain of rubber composites with untreated PALF, (**b**) stress-strain of rubber composites with treated PALF, (**c**) stress-strain of rubber composites with untreated HF, (**d**) stress-strain of rubber composites with treated HF, and (**e**) Young's modulus of rubber composites.

Table S1. Formulation of rubber compound with and without natural fibres.

Formulas	Ingradient (phr)								
	**NR STR20**	**Steric acid**	**ZnO**	**N330**	**CBS**	**Sulphur**	**Silane 69**	**PALF**	**HF**
Control	100	1	3	40	1	2	-	-	-
NR/PALF 25 phr	100	1	3	40	1	2	-	25	-
NR/PALF 50 phr	100	1	3	40	1	2	-	50	-
NR/PALF 75 phr	100	1	3	40	1	2	-	75	-
NR/Si 69/PALF 25 phr	100	1	3	40	1	2	2.5	25	-
NR/Si 69/PALF 50 phr	100	1	3	40	1	2	5.0	50	-
NR/Si 69/PALF 75 phr	100	1	3	40	1	2	7.5	75	-
NR/HF 25 phr	100	1	3	40	1	2	-	-	25
NR/HF 50 phr	100	1	3	40	1	2	-	-	50
NR/HF 75 phr	100	1	3	40	1	2	-	-	75
NR/Si 69/HF 25 phr	100	1	3	40	1	2	2.5	-	25
NR/Si 69/HF 50 phr	100	1	3	40	1	2	5.0	-	50
NR/Si 69/HF 75 phr	100	1	3	40	1	2	7.5	-	75

Note: phr means part per hundred parts of rubber.

Table S2. The Mooney–Rivlin constants (C_1 and C_2) of the sample

Sample	Test	$2C_1$	C_1	$2C_2$	C_2	C_2 / C_1
Control	Extension	241.32	120.65	497.40	248.70	2.06
NR/PALF 25 phr	Extension	479.38	239.69	955.27	477.64	1.99
NR/PALF 50 phr	Extension	2796.30	1398.15	7633.00	3816.50	2.73
NR/PALF 75 phr	Extension	2003.20	1001.60	5132.1	2566.05	2.56
NR/Si 69/PALF 25 phr	Extension	7447.10	3723.55	14813	7406.5	1.99
NR/Si 69/PALF 50 phr	Extension	8144.82	4072.41	28293.90	14146.95	3.47
NR/Si 69/PALF 75 phr	Extension	2524.00	1262.15	6292.7	3146.35	2.49
NR/HF 25 phr	Extension	1340.20	222.41	3217.1	1608.55	2.40
NR/HF 50 phr	Extension	1031.10	515.55	3132.5	1566.25	3.04
NR/HF 75 phr	Extension	1168.00	584.10	2948	1474	2.52
NR/Si 69/HF 25 phr	Extension	2614.90	649.00	2716.00	1358.00	1.68
NR/Si 69/HF 50 phr	Extension	1626.30	2925.40	3812.8	1906.40	2.34
NR/Si 69/HF 75 phr	Extension	5850.80	1307.45	10803.00	5401.50	1.85

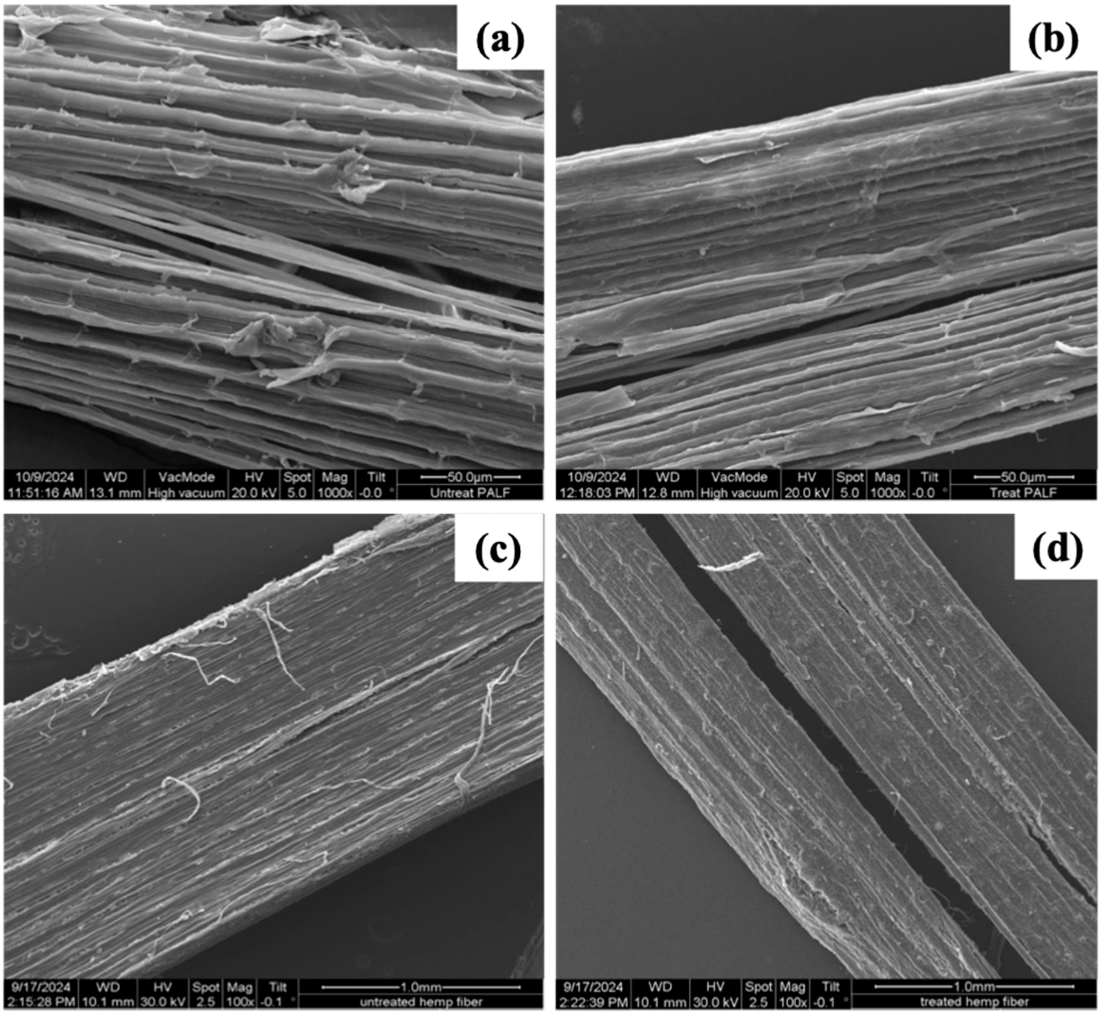

Figure S4. SEM images of bundle of natural fibres: (a) untreated PALF, (b) NaOH and Silane 69 treated PALF, (c) untreated HF, and (d) NaOH and Silane 69 treated HF.

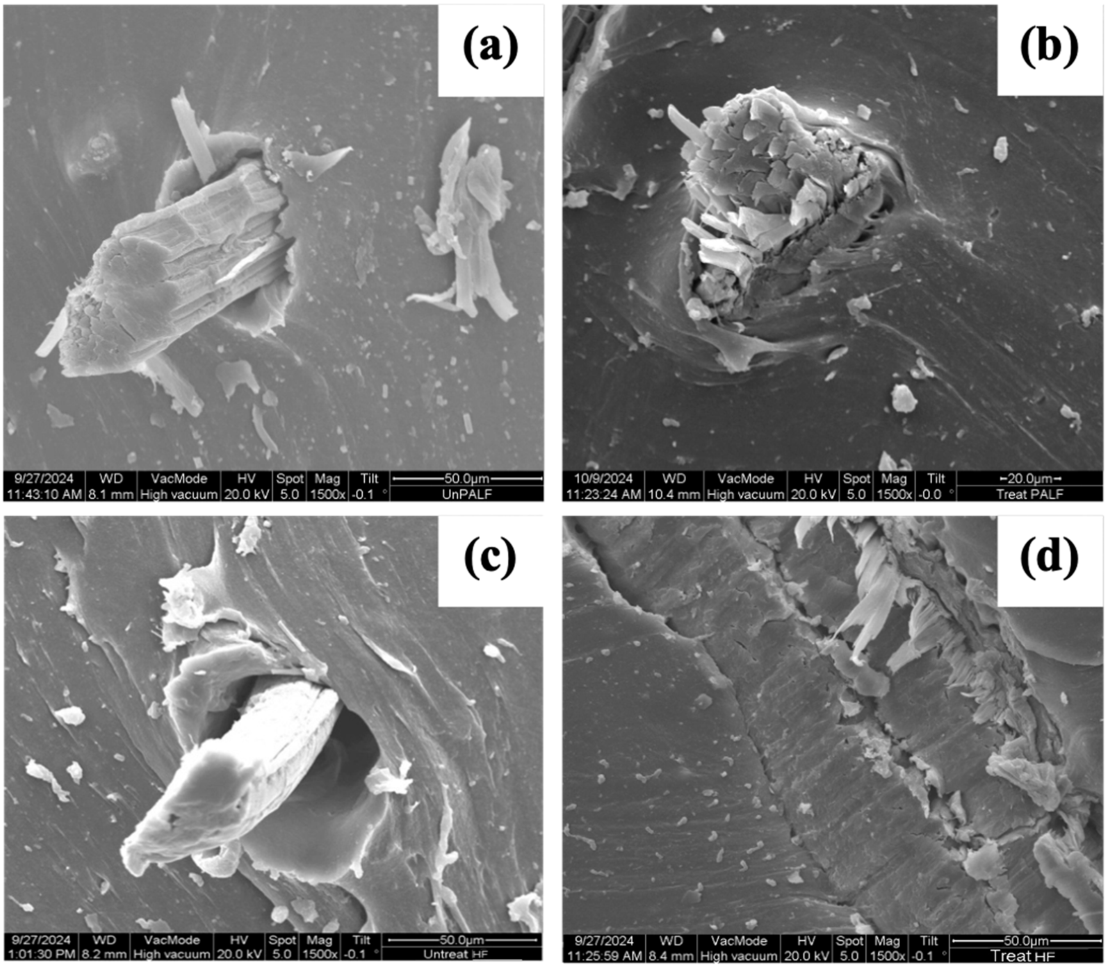

Figure S5. Fractured surface of rubber composites with PALF and HF at 50 phr after tensile strength testing: (a) untreated PALF, (b) PALF treated with NaOH and Silane 69 (c) untreated HF, and (d) HF treated with NaOH and Silane 69.

Table S3. The tensile properties of the sample

Sample	Young's modulus (MPa)	Tensile strength (MPa)	Elongation at break (%)
Control	0.35^{a}	4.4^{e}	534^{i}
NR/PALF 25 phr	3.97^{b}	1.87^{b}	175^{b}
NR/PALF 50 phr	5.64^{f}	1.97^{b}	173^{b}
NR/PALF 75 phr	5.92^{f}	1.77^{b}	184^{b}
NR/Si 69/PALF 25 phr	4.14^{c}	3.69^{d}	254^{c}
NR/Si 69/PALF 50 phr	5.82^{f}	5.75f	453^{g}
NR/Si 69/PALF 75 phr	6.01^{f}	2.53^{c}	266^{d}
NR/HF 25 phr	3.74 ^{b}	1.79^{b}	384^{f}
NR/HF 50 phr	5.04^{d}	2.02^{b}	500^{h}
NR/HF 75 phr	5.24^{e}	1.35^{a}	82^{a}
NR/Si 69/HF 25 phr	4.08^{c}	2.55^{c}	323^{e}
NR/Si 69/HF 50 phr	5.42^{f}	5.61^{c}	601^{j}
NR/Si 69/HF 75 phr	5.58^{f}	1.62^{a}	173^{b}

$^{a-i}$: The one-way analysis indicates statistically significant variations among the various letters Young's modulus, Tensile strength and Elongation at break at a 95% significance level ($p < 0.05$).
